# Enzymatic response to cadmium by *Impatiens glandulifera*: A preliminary investigation

**DOI:** 10.1016/j.bbrep.2021.100936

**Published:** 2021-02-09

**Authors:** Stephanie Coakley, Gary Cahill, Anne-Marie Enright, Brian O'Rourke, Carloalberto Petti

**Affiliations:** Department of Science and Health, EnviroCORE, Institute of Technology Carlow, Kilkenny Road, R93 V960, Carlow, Ireland

**Keywords:** Catalase (CAT), Glutathione S-transferase (GST), Ascorbate peroxidase (APX), Superoxide dismutase (SOD), Proline, Photosynthetic pigments

## Abstract

This paper aims to develop our understanding of the effect of cadmium (Cd) on *Impatiens glandulifera*, a recently identified potential Cd hyperaccumulator. *Impatiens glandulifera* plants were exposed to three concentrations of Cd (20, 60 and 90 mg/kg) and were sampled at two timepoints (one and seven days) to investigate the stress response of *I. glandulifera* to Cd. Cd can induce oxidative stress in plants, triggering overproduction of reactive oxygen species (ROS). The level of activity of catalase (CAT) and ascorbate peroxidase (APX), two crucial antioxidant enzymes responsible for detoxifying ROS, were found to increase in a concentration dependent manner. Though there was no change observed in the level of superoxide dismutase (SOD) activity, the activity of glutathione S-transferase (GST), involved in detoxifying and sequestering Cd, increased after exposure to Cd. Cd did not appear to impact the levels of proline and photosynthetic pigments, indicating the plants weren't stressed by the presence of Cd. These results suggest that the rapid response observed in enzyme activity aid the efficacious mitigation of the toxic effects of Cd, preventing significant physiological stress in *I. glandulifera*.

## Highlights

• *Impatiens glandulifera* display an enhance tolerance to Cadmium.
• An early response in a Catalase and Peroxidase ascorbate mediates Cadmium tolerance.
• No impact on stress indicators were shown by *Impatiens glandulifera* even after 7 days.
• SOD was found to be not involved in the early response to Cadmium.

## 1 Introduction

Augmenting levels of Cd contamination, caused by anthropogenic activity, is a worldwide concern due to the toxic nature of Cd [[1], [2], [3], [4]]. Levels as low as 2.5 mg/kg Cd can cause physiological damage in plants, induce reactive oxygen species (ROS) and lead to cell death [1,5]. However, some plants have the ability to tolerate Cd, to grow healthily while accumulating high levels of Cd in their tissues [1,6,7].

Phytoaccumulation is a sustainable, low-cost option which involves the use of plants to stabilise or remove Cd contamination. The discovery of highly tolerant hyperaccumulator plants has enhanced the efficacy of phytoremediation, making it a more viable option [8,9]. However, the use of hyperaccumulator plants is limited as many are low biomass, slow growing plants and several effective hyperaccumulators are crop species [8,10]. In order to broaden the application of phytoremediation, we need to develop our understanding of the physiological mechanisms involved [1].

This paper is a continuation of our previous research which presented *Impatiens glandulifera* as a new potential Cd hyperaccumulator [11]. *Impatiens glandulifera* possesses many of the qualities of an ideal Cd hyperaccumulator. It is a fast growing, easy to harvest plant which is resistant to disease and pests and can be grown under a variety of conditions.

In our previous research, we highlighted the ability of *I. glandulifera* to accumulate high levels of Cd and to translocate Cd into its aboveground tissues. To be considered a Cd hyperaccumulator, plants must be able to accumulate 100 mg/kg Cd in its stems; this is approximately 100 times the level of Cd which would be found in the tissues of a nonhyperaccumulator species [5]; *I. glandulifera* accumulated between 276 and 1562 mg/kg Cd in its stems [11]. The physiological measurements taken from our phytoremediation and germination trials indicated an impressive tolerance for the toxicity of Cd. The plants grew normally in the presence of up to 150 mg/kg of Cd, with no significant difference in the height or biomass of the plants. Seeds of *I. glandulifera* were able to survive exposure to extremely high concentrations of Cd, with seeds germinating normally in the presence of 1000 mg/kg Cd [11]. As *I. glandulifera* did not show visible signs of stress, this paper seeks to expand on our previous observations of hypertolerance by examining common biochemical markers of stress in plants that is proline and photosynthetic pigments [12,13].

Plants are known to vary in their tolerance, accumulation, ability to translocate and partition Cd [6,7,14]. This stems from the efficacy of the mechanisms they employ in response to Cd.

A major route of detoxification of Cd involves its chelation to thiol-containing molecules, such as glutathione, phytochelatins or metallothioneins [6,15]. The Cd complexes formed via chelation are less toxic and can be more easily mobilised to storage sites, such as vacuoles [16,17]. The subcellular distribution of Cd plays an important role in its detoxification and tolerance in many species, including hyperaccumulators [2,16,18]. Our previous research indicated that *I. glandulifera* may use compartmentalisation as a mechanism of tolerance. Therefore, in this paper the activity of glutathione s-transferase (GST), which catalyses the conjugation of Cd to glutathione, is investigated.

One of the most utilized and effective mechanisms of tolerance in plants is the activation of the antioxidant defense system [19]. Antioxidant enzymes, such as superoxide dismutase (SOD), catalase (CAT) and those involved in the ascorbate-glutathione cycle, infer tolerance to plants by detoxifying excess ROS, which are produced in response to Cd [2,6,16]. This research assesses the activity of SOD, CAT and APX to ascertain if antioxidant enzymes are employed by *I. glandulifera* as a mechanism of Cd tolerance.

## 2 Materials and methods

### 2.1 Plant materials and Cd treatments

This experiment was set up in the same manner as our previous bioaccumulation experiment (see Coakley et al. [11]). Therefore, the *I. glandulifera* plants utilized were the same age (circa two months old and 30 cm tall) and were planted into Westland topsoil/multipurpose compost (pH 5.8, 2.7 mg/kg organic carbon, 74 mg/kg nitrogen, 39.3 mg/kg phosphorus and 29.4 mg/kg potassium). Three seedlings were planted per pot in 500 g (±25 g) of compost (dry weight) in polyethylene pots. The control had three pots (nine plants), while Cd-20, Cd-60 and Cd-90 each had six pots (eighteen plants). After the seedlings were planted, they were spiked with an aqueous solution of CdCl_2_ to give final soil concentrations of 20 mg/kg, 60 mg/kg and 90 mg/kg Cd. The control plants (0 mg/kg Cd) were not spiked with CdCl_2_. The plants were harvested after two time points: one- and seven-days post-spiking. The control plants (0 mg/kg Cd) were harvested alongside the plants which had been exposed to Cd for 24 h.

The pots were irrigated twice a day with circa 50 mL of water. The plants were grown in a temperature-controlled glasshouse at a 22 °C ± 2 °C constant temperature in the Institute of Technology Carlow, Ireland. A 16-h light/8-h dark cycle was employed with a light intensity of 1725 μmol/s/m^2^ maintained for the sixteen light hours. The roots and shoots (stems and leaves) were harvested and the plant material from each pot was pooled, giving rise to three pooled samples per treatment, and stored at – 80 °C. Total trial length from germination to harvest was circa 3 months. All chemicals were sourced from Fisher Scientific Ireland Limited (Dublin, Ireland).

### 2.2 Protein extraction and estimation

Plant samples (2 g fresh weight) were ground in liquid nitrogen and homogenized in 20 mL of phosphate buffer (100 mM pH 7.5 potassium phosphate with 1 mM ethylenediaminetetraacetic acid disodium salt and 5% (w/v) polyvinylpolypyrrolidone). The samples were centrifuged at 4 °C at 10,000 g for 30 min. All steps were carried out on ice and the supernatant was stored at −80 °C prior to being used for enzyme assays (CAT, SOD, GST, APX) [20,21]. The Bradford protein assay was utilized to quantify the extracted protein present in the samples [22].

### 2.3 Enzymatic activity assays

Superoxide dismutase (SOD, EC 1.15.1.1) activity was determined on the basis of the reduction of nitro blue tetrazolium chloride (NBT) following Dhindsa *et al*., [23]. One unit of SOD activity (U) is defined as the amount of enzyme required to cause a 50 % inhibition of NBT photoreduction rate. Ascorbate peroxidase (APX, EC 1.11.1.11) activity was measured according to Jiang and Zhang [20] by following the rate of disappearance of H_2_O_2_ at 290 nm over 1 min. Catalase (CAT, EC 1.11.1.6) activity was measured in accordance with Georgiadou et al. [24] by following the rate of disappearance of H_2_O_2_ at 240 nm over 1 min. Glutathione S-transferase (GST, EC 2.5.1.18) activity was determined using chloro-2,4-dinitrobenzene (CDNB) as a substrate following Habig and Jakoby [25] by measuring absorbance at 340 nm every minute for 3 min. The enzyme activity of SOD, APX, CAT and GST were expressed as μmol of product formed per min per mg of protein.

### 2.4 Photosynthetic pigment analysis

The photosynthetic pigments were extracted from 0.2 g (fresh weight) of leaves and ground in 5 mL of 80 % acetone at 4 °C. The extracts were filtered using grade 1 Whatman equivalent filter paper. The absorbance of the samples were read at 470 nm (carotenoids), 649 nm (Chlorophyll b) and 665 nm (Chlorophyll a). Acetone (80 %) was used as a control [4,26]. Calculations were completed according to Lichtenthaler and Buschmann [27].

### 2.5 Proline content quantification

This method was adapted from Sun and Hong [21] and Shabnam *et al*., [28]. Plant material (0.5 g fresh weight) was triturated in liquid nitrogen and homogenized with 10 mL methanol:chloroform:water (12:5:1). The solution was centrifuged at 5000×*g* for 5 min. The centrifuged extract (1 mL) was mixed with 2 mL of 1.25 % ninhydrin in glacial acetic acid and incubated at 100 °C for 30 min. The absorbance of the proline-ninhydrin condensation product in the reaction mixture was measured at 508 nm. The quantity of proline in the samples was calculated from a standard graph.

### 2.6 Data analysis

To assess if the level of enzyme activity, or indeed the concentration of proline or photosynthetic pigments, was significantly different in the roots and shoots of control plants versus Cd-treated plants, Kruskal Wallis with pairwise comparisons was utilized. Statistical significance was defined at an alpha value of 0.05.

## 3 Results

### 3.1 Antioxidant enzyme response to Cd

Exposure to Cd for one day was associated with a substantial increase in the level of enzyme activity in the case of APX, CAT and GST (see Table 1). The level of activity of APX, CAT and GST was found to increase in a concentration dependent manner and decreased over time. Indeed, in some cases the level of antioxidant activity returned to control levels after seven days. Expectedly, the roots of *I. glandulifera*, which were directly exposed to Cd, were typically found to host higher levels of antioxidant activity than the shoots. In particular, the level of APX and CAT activity in the roots was substantially higher than the shoots (on average, 77.0 ± 29.3 % and 71.2 ± 5.6 % higher in roots than shoots for APX and CAT, respectively). The level of GST activity was, on average, 22.9 ± 8.1 % greater in the roots than the shoots, with one exception-the level of GST activity in the shoots of plants exposed to 90 mg/kg Cd for seven days were higher than the level of GST activity in the roots. The highest increment in enzyme activity of plants exposed to Cd versus control plants was observed for CAT (61.5 ± 14.6 % and 67.8 ± 11.6 % higher in roots and shoots of Cd-treated plants). Moreover, the shoots of plants exposed to 60 mg/kg and 90 mg/kg Cd for one day were found to have significantly higher levels of CAT than the control (See Table 1, p ≤ 0.05). Iterating the pattern observed for GST, the increase of APX activity was more pronounced in the roots than the shoots of plants exposed to Cd than in control plants (43.6 ± 23.3 % and 31.4 ± 10.4 % higher in roots and shoots of Cd-treated plants). While the difference was non-significant between control plants and the 20 mg/kg Cd treatments, there was a significant increase in the activity of APX in the roots of plants exposed to 60 mg/kg Cd for one day and 90 mg/kg Cd for both one and seven days (See Table 1, p ≤ 0.05).

**Table 1:** Response of enzyme activity to Cd in *Impatiens glandulifera*. Enzyme activity presented as averages ± SD (n = 3). Values with different lowercase letters represent statistically significant differences between Cd treatments and control for an enzymes and tissue type (*i.e.* either roots or shoots). Statistical significance is defined at the alpha value of ≤0.05.

Cd mg/kg	*SOD activity (μmol product/min/mg protein)*		*APX activity (μmol product/min/mg protein)*		*CAT activity (μmol product/min/mg protein)*		*GST activity (μmol product/min/mg protein)*	
	Roots	Shoots	Roots	Shoots	Roots	Shoots	Roots	Shoots
***Control (0)***	9.906 ± 2.20545	64.036 ± 49.13735 a	0.18 ± 0.01353 a	0.025 ± 0.00081 a	0.007 ± 0.00106 a	0.002 ± 0.00014 a	0.030 ± 0.00108 a	0.010 ± 0.00005 a
***20*** ***Day 1***	7.623 ± 1.90612 a	45.297 ± 26.19474 a	0.398 ± 0.00309 ab	0.047 ± 0.00067 a	0.015 ± 0.00012 a	0.005 ± 0.00022 a	0.076 ± 0.00086 a	0.014 ± 0.00005 b
***60*** ***Day 1***	36.832 ± 30.98503 a	200.626 ± 41.72306 a	0.409 ± 0.06307 b	0.054 ± 0.00492 a	0.026 ± 0.00073 a	0.007 ± 0.00033 a	0.086 ± 0.00325 a	0.014 ± 0.00006 b
***90*** ***Day 1***	14.149 ± 1.89659 a	159.097 ± 11.01593 a	0.497 ± 0.01977 b	0.063 ± 0.00250 a	0.045 ± 0.00017 a	0.009 ± 0.00019 a	0.094 ± 0.00279 a	0.013 ± 0.00004 b
***20*** ***Day 7***	29.510 ± 6.53793 a	172.232 ± 48.36875 a	0.188 ± 0.00301 ab	0.015 ± 0.00036 a	0.013 ± 0.00172 a	0.004 ± 0.00008 a	0.025 ± 0.00387 a	0.012 ± 0.00030 ab
***60*** ***Day 7***	6.176 ± 4.77187 a	90.098 ± 6.93309 a	0.365 ± 0.06249 ab	0.023 ± 0.00003 a	0.015 ± 0.00131 a	0.005 ± 0.00010 a	0.015 ± 0.00098 a	0.012 ± 0.00022 ab
***90*** ***Day 7***	33.261 ± 11.34957 a	164.435 ± 59.87420 a	0.445 ± 0.02000 b	0.023 ± 0.00018 a	0.019 ± 0.00127 a	0.007 ± 0.00006 a	0.010 ± 0.00105 a	0.011 ± 0.00092 ab

Notably, while the level of GST activity in Cd-treated plants was initially elevated compared to control plants, there was a prominent decrease in GST activity over time. The GST activity in the roots of *I. glandulifera* plants exposed to Cd was 64 ± 3.7 % higher than control plants after one day and 44.8 ± 25.6 % lower than control plants after seven days (see Table 1). Furthermore, there was a significant increase in GST activity in the shoots of plants exposed to 20, 60 and 90 mg/kg Cd for one day compared to control plants (p ≤ 0.05, Table 1). However, this increment in GST activity was approximately halved after seven days (29.1 ± 3.4 % and 16.7 ± 6.65 %)

Interestingly, there was no difference observed in the levels of SOD between treatments and controls at either timepoint (one and seven days). Despite the above, across all Cd treatments and control, the level of SOD activity was found to be higher in shoots than roots (Table 1).

### 3.2 Effect of Cd on proline and photosynthetic pigments: indicators of plant stress

Interestingly, Cadmium did not appear to have an impact on proline levels in *I. glandulifera* at either of the timepoints tested (Day 1 and 7). Similar to SOD, there was little difference observed in the level of proline across all treatments. The level of proline was consistently higher in the shoots than the roots.

The concentration of chlorophyll a, chlorophyll b and carotenoids in plants exposed to Cd were not found to differ significantly from control plants at either timepoint. The levels of chlorophyll *a* and *b* remained constant across all treatments, including control, with one exception-a peak in the concentration of chlorophyll b was observed in plants exposed to 90 mg/kg Cd for one day (see Table 2). Interestingly, a concomitant decrease in the concentration of carotenoids was observed in plants exposed to 90 mg/kg Cd for one day.

**Table 2:** Response of stress indicators to Cd in *I. glandulifera*. Photosynthetic pigments and proline concentrations presented as averages ± SD (n = 3). Statistical significance is defined at p ≤ 0.05.

	Photosynthetic pigments (mg/g protein)		Proline (mg/g protein)		
	Chlorophyll a	Chlorophyll b	Carotenoids	Roots	Shoots
***Control***	0.540 ± 0.033	0.361 ± 0.064	0.081 ± 0.017	57.81 ± 22.16	215.70 ± 27.73
**20 mg/kg Cd *Day 1***	0.569 ± 0.023	0.498 ± 0.197	0.053 ± 0.047	58.35 ± 18.49	209.85 ± 26.98
**60 mg/kg Cd *Day 1***	0.561 ± 0.009	0.483 ± 0.184	0.045 ± 0.039	47.50 ± 13.16	209.58 ± 25.23
**90 mg/kg Cd *Day 1***	0.569 ± 0.022	0.779 ± 0.121	0.000 ± 0.000	24.17 ± 9.06	269.26 ± 1.15
**20 mg/kg Cd *Day 7***	0.548 ± 0.044	0.386 ± 0.094	0.079 ± 0.019	46.42 ± 6.78	170 ± 4.60
**60 mg/kg Cd *Day 7***	0.548 ± 0.039	0.357 ± 0.079	0.084 ± 0.009	52.93 ± 24.92	165.78 ± 32.97
**90 mg/kg Cd *Day 7***	0.564 ± 0.013	0.498 ± 0.209	0.046 ± 0.041	67.11 ± 34.53	190.20 ± 6.91

## 4 Discussion

This paper’ research design aimed to capture the early and late enzymatic responses of *I. glandulifera* to Cd, to characterise some of the enzymes which have the potential to contribute to the Cd tolerance we observed in our previous research [11]. Cd is known to elicit a variable response from plants [6,14,29,30] and different plants are known to utilise different mechanisms of tolerance [2,16,29]. The effective Cd tolerance of a variety of species, including the invasive perennial *Phragmites australis* and the Cd hyperaccumulator *Arabidopsis thaliana*, has been associated with the prompt activation of antioxidant enzymes and vacuolar sequestration of Cd via GST to alleviate the negative effects of Cd [6,[31], [32], [33]]. Our results suggest that *I. glandulifera* employs a similar strategy to achieve Cd-tolerance.

Our results captured the early and short-term effects of Cd on *I. glandulifera*, indicating that *I. glandulifera's* response to Cd-induced stress is rapid, with levels of APX, CAT and GST activities rising sharply after one day. A subsequent decline in antioxidant activity was observed after seven days, with control-levels of activity observed in some cases. Interestingly, the activities of antioxidant enzymes APX and CAT, which play a similar role in detoxifying ROS [2,34,35], were found to significantly increase in different parts of *I. glandulifera*. Evidence from other plant species shows that the response of antioxidant enzymes is different in leaves and roots [6,14,36]; in this study, the activity of APX was found to significantly increase in the roots, whereas the activity of CAT was found to significantly increase in shoots.

Our previous research, which visualized Cd in the roots, stems and leaves of *I. glandulifera,* suggested that compartmentalisation appeared to play a role in the Cd-tolerance of *I. glandulifera* [11]. The results from this study, which found that the activity of GST significantly increased in the shoots of *I. glandulifera*, appear to support this theory.

The results from our earlier bioaccumulation and germination trials indicated that *I. glandulifera* is hypertolerant of Cd [11]. The results presented here concur with the visual observations and physiological measurements taken in our previous trials. As expected, the biochemical markers which can provide an indication of stress in plants did not show any significant differences between controls and treatments. The maintenance of control levels of chlorophyll *a* and *b* suggest that the plants defence system provides sufficient tolerance to essential cell components to allow normal functioning [[37], [38], [39]]. Similar to its effect on enzyme activities, Cd is known to have a differential effect on the concentration of proline-which can be a useful indicator of Cd stress in plants [7,12,13]. The maintenance of control levels of proline suggests that the stress threshold has not been exceeded in this trial. Furthermore, proline is an osmolyte which is sometimes involved in scavenging ROS [7,38,39]; the proline results therefore also imply that proline is not involved in the defence system of *I. glandulifera.*

The results presented in this paper provide the basis for future research which will further develop our comprehension of the mechanisms responsible for imparting Cd-tolerance to *I. glandulifera*. We postulate that a sampling point within 24 h of spiking may reveal significant increases in SOD activity in *I. glandulifera*. Furthermore, based on this research, we recommend a more detailed compartmentalisation study, examining other chelating agents, such as phytochelatins. Further studies of the tolerance mechanism of *I. glandulifera* may have applications in enhancing phytoremediation or in devising means to protect other plant species from Cd-stress.

## Author contributions

Conceptualization, C.P. and S.C.; methodology, C.P. and S.C.; validation, S.C.; formal analysis, S.C. and C.P.; investigation, S.C.; resources, S.C.; data curation, S.C.; writing—original draft preparation, S.C.; writing—review and editing, C.P., G.C., B.O. and A.-M.E.; visualization, S.C., C.P. and G.C.; supervision, C.P., G.C., A.-M.E. and B.O.; project administration, C.P., S.C., G.C.; funding acquisition, C.P.

## Funding

This research was funded by President's Research Fellowship Scholarship, grant number PES1219, from the Institute of Technology Carlow.

## Declaration of competing interest

The authors declare no conflict of interest.
